# Bladder Adenocarcinoma in a Constellation of Multiple Site Malignancies: An Unusual Case and Systematic Review

**DOI:** 10.3390/diagnostics14222510

**Published:** 2024-11-09

**Authors:** Daniel Porav-Hodade, Raul Gherasim, Andrada Loghin, Bianca Lazar, Ovidiu Simion Cotoi, Mihail-Alexandru Badea, Mártha Orsolya Katalin Ilona, Ciprian Todea-Moga, Mihai Dorin Vartolomei, Georgescu Rares, Nicolae Crisan, Ovidiu Bogdan Feciche

**Affiliations:** 1 Department of Urology, George Emil Palade University of Medicine, Pharmacy, Science, and Technology of Târgu Mureș, 540139 Târgu Mureș, Romania; daniel.porav-hodade@umfst.ro (D.P.-H.); orsolya.martha@umfst.ro (M.O.K.I.); 2 Department of Urology, Clinical County Hospital Mures, 540136 Târgu Mures, Romania; raul.dumitru-gherasim@umfst.ro; 3 Department of Pathophysiology, George Emil Palade University of Medicine, Pharmacy, Science, and Technology of Târgu Mureș, 540139 Târgu Mureș, Romania; andrada.loghin@umfst.ro (A.L.); ovidiu.cotoi@umfst.ro (O.S.C.); 4 Department of Pathophysiology, Clinical County Hospital Mures, 540136 Târgu Mures, Romania; ohii.bianca@yahoo.com; 5 Department of Dermatology, George Emil Palade University of Medicine, Pharmacy, Science, and Technology of Târgu Mureș, 540139 Târgu Mureș, Romania; mihail.badea@umfst.ro; 6 Department of Dermatology, Clinical County Hospital Mures, 540136 Târgu Mures, Romania; 7 Department of Cell and Molecular Biology, George Emil Palade University of Medicine, Pharmacy, Science, and Technology of Târgu Mureș, 540139 Târgu Mureș, Romania; mdvartolomei@yahoo.com; 8 Department of General Surgery, George Emil Palade University of Medicine, Pharmacy, Science, and Technology of Târgu Mureș, 540139 Târgu Mureș, Romania; rares.georgescu@umfst.ro; 9 Department of General Surgery, Clinical County Hospital Mures, 540136 Târgu Mures, Romania; 10 Department of Urology, Iului Hatieganu University of Medicine and Pharmacy, 400012 Cluj-Napoca, Romania; 11 Department of Urology, Faculty of Medicine and Pharmacy, University of Oradea, 410087 Oradea, Romania; feciche.bogdanovidiu@didactic.uoradea.ro; 12 Department of Urology, Emergency County Hospital Oradea, 410169 Oradea, Romania

**Keywords:** multiple tumors, malignant melanoma, primary malignancies, secondary malignancies, genitourinary malignancies, metachronous, synchronous, adenocarcinoma, risks factors, multicancer early detection test

## Abstract

Background and Objectives: Multiple primary malignant tumors represent a small percentage of the total number of oncological cases and can involve either metachronous or synchronous development and represent challenges in diagnosis, staging, and treatment planning. Our purpose is to present a rare case of bladder adenocarcinoma in a female patient with multiple primary malignant tumors and to provide systematic review of the available literature. Materials and Methods: A 67-year-old female patient was admitted with altered general condition and anuria. The past medical history of the patient included malignant melanoma (2014), cervical cancer (2017), colon cancer (2021), obstructive anuria (2023), and liver metastasectomy (2023). Transurethral resection of bladder tumor was performed for bladder tumors. Results: Contrast CT highlighted multiple pulmonary metastases, a poly nodular liver conglomerate, retroperitoneal lymph node, II/III grade left ureterohydronephrosis, and no digestive tract tumor masses. The pathological result of the bladder resection showed an infiltrative adenocarcinoma. Conclusions: The difference between primary bladder adenocarcinoma tumor and metastatic colorectal adenocarcinoma is the key for the future therapeutic strategy. Identification and assessment of risk factors such as viral infection, radiotherapy, chemotherapy, smoking, and genetics are pivotal in understanding and managing multiple primary malignant tumors. Personalized prevention strategies and screening programs may facilitate the early detection of these tumors, whether synchronous or metachronous. The use of multicancer early detection (MCED) blood tests for early diagnosis appears promising. However, additional research is needed to standardize these techniques for cancer detection.

## 1. Introduction

Multiple primary malignant (MPM) tumors represent a small percentage of the total number of oncological cases. This percentage is inversely proportional to the number of primary malignant tumors [1].

Multiple primary cancers can involve either metachronous or synchronous development [2]; Warren and Gates were the ones who made this division of multiple primary tumors [3].

Synchronous primary malignancy (SPM) is characterized by the concurrent occurrence of multiple primary tumors in the same patient, where the second primary cancer is diagnosed within 6 months of the primary cancer.

Metachronous primary tumors (MPM) are secondary primary tumors that develop in a different organ or location from the primary cancer, and the second tumor may be detected 6 months afterwards [4].

Beyond this definition, more than two primary malignancies occurring at different times is defined as metachronous multiple primaries [2].

The presence of synchronous or metachronous tumors can pose challenges in diagnosis, staging, and treatment planning. It requires a comprehensive evaluation to determine the extent of disease and the appropriate management strategies.

We must also consider the possibility of secondary tumor metastases, which will increase the complexity of managing these patients, particularly in terms of diagnostic strategies and, most importantly, therapeutic approaches.

## 2. Case Presentation

On 9 February 2024, a 67-year-old female patient was admitted to the Urology Department of the Mures County Clinical Hospital for a slightly altered general condition, anuria, and altered renal samples (creatinine = 5.8 mg/dL, K = 7.1 mmol/L), with BMI = 32.

The patient smoked for about 30 years, but she gave up this habit 10 years ago.

The patient’s past medical history was extremely generous.

**Figure 1:**
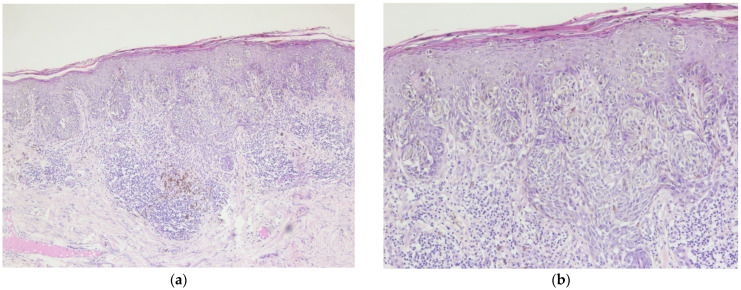
Invasive malignant melanoma, superficial spreading subtype: (**a**) Hematoxylin and eosin staining (H&E), 5× magnification. (**b**) Superficial spreading melanoma with haphazardly distributed atypical melanocytes present as single cells and nests at all levels of the epidermis, H&E, 10× magnification.

**Figure 2:**
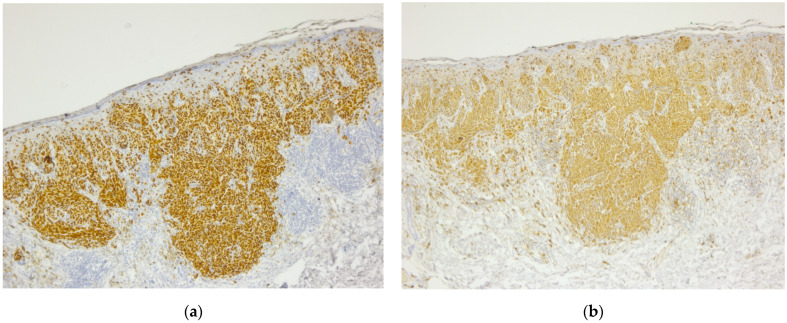
Invasive malignant melanoma: (**a**) SOX10 immunostain highlights nuclear positivity of malignant melanocytes, 5× magnification. (**b**) S100 immunostain highlights positivity of malignant melanocytes, 5× magnification.

**Figure 3:**
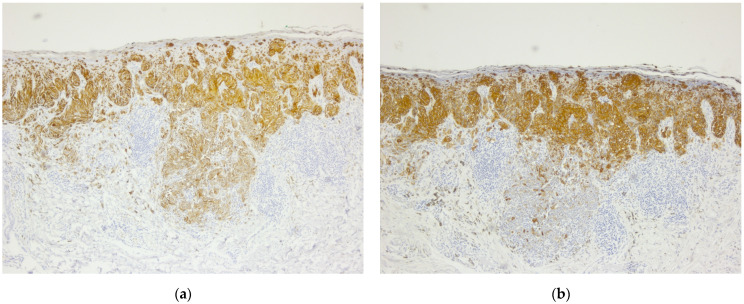
Invasive malignant melanoma: (**a**) Melan A immunohistochemical stain, a marker of melanocytic differentiation, highlights intraepithelial pagetoid spread as well as malignant melanocytes at the epithelial–connective tissue interface and in the superficial connective tissue, 5× magnification. (**b**) HMB45 immunostain highlights cytoplasmic positivity of all melanocytes, including deep dermal nests of atypical melanocytes, 5×.

In 2014, the patient presented malignant melanoma, the tumor at the skin level being <0.75, for which surgical excision was performed (July 2014) within oncological safety limits. The pathological result was invasive malignant melanoma of the superficial spreading subtype (Figure 1, Figure 2 and Figure 3).

**Figure 4:**
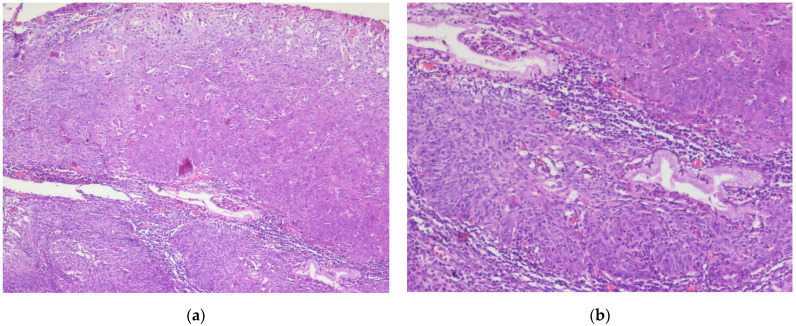
Squamous cell carcinoma of the cervix: (**a**) Non-keratinizing squamous cell carcinoma, squamous cells in islands infiltrating deeper tissue with individual cell keratinization but lack epithelial pearls, H&E, 5× magnification. (**b**) Squamous cell carcinoma of the cervix, non-keratinizing type. Malignant squamous cells have abundant eosinophilic cytoplasm, distinct cell borders, and individual cell keratinization, H&E, 10× magnification.

In March 2017, the patient was diagnosed with stage IA2 cervical cancer, for which internal radiation therapy was practiced using the Varian GammaMedPlus iX technique, followed in the same year (October 2017) by a radical hysterectomy. The pathological assessment revealed the presence of cervical squamous cell carcinoma (Figure 4).

Enhanced computed tomography (CT) follow-up of the abdomen and pelvis, performed annually until 2019, did not show any local recurrences or distant metastases. Subsequently, the patient did not return for oncological follow-up surveillance protocol.

**Figure 5:**
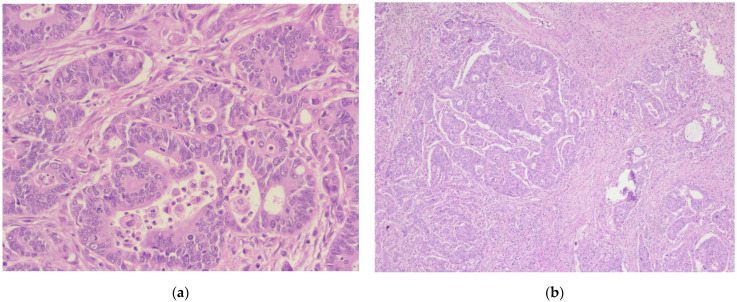
Sigmoid colon adenocarcinoma: (**a**) Central comedonecrosis: necrotic debris inside the neoplastic gland, H&E, 10× magnification. (**b**) Hematoxylin and eosin (H&E) stained sigmoid colon showing grade two, moderately differentiated adenocarcinoma, 5× magnification.

In 2021, the patient had multiple episodes of rectal bleeding that were initially overlooked. Later that year (August), a colonoscopy and subsequent biopsy confirmed the diagnosis of sigmoid colon adenocarcinoma (Figure 5). A partial colectomy was performed for stage II sigmoid cancer (November 2021), followed by five sessions of adjuvant chemotherapy (fluoropyrimidine-based).

In 2022, the patient presented an episode of obstructive anuria. The CT scan performed showed bilateral ureterohydronephrosis due to bilateral stenosis of the last part of the ureter, interpreted as the consequence of brachytherapy for cervical cancer.

The same CT examination highlighted the presence of tumors located in the liver, which was interpreted as liver metastases. The replacement of the bilateral ureteral stents 7 Ch was practiced for a period of 12 months and was followed by the normalization of the renal tests.

**Figure 6:**
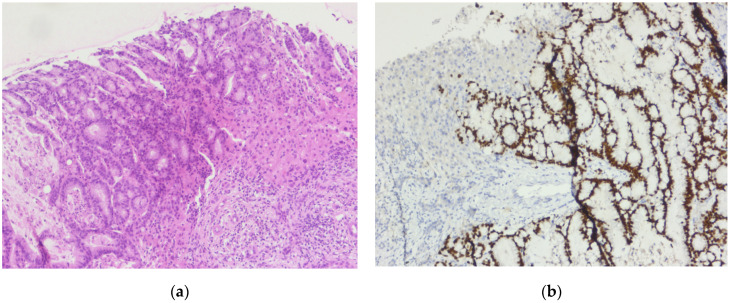
Liver metastasis of the sigmoid colon cancer: (**a**) Tumor proliferation composed of irregular, crowded glands, lined by a stratified columnar epithelium with marked cytonuclear atypia, with hyperchromatic and elongated nuclei, H&E, 10× magnification. (**b**) CDX-2 immunostain highlights positivity within the tumor cells, 10× magnification.

In February 2023, the metastasectomy of the liver tumor was performed. The pathological examination revealed a liver metastasis of a tumor with a digestive starting point (colon) (Figure 6). The patient did not receive (refuse) any further adjuvant oncological treatment.

Upon admission to the hospital, the ultrasound evaluation revealed bilateral ureterohydronephrosis despite the proper positioning of both stents. Given the time elapsed since the stents were inserted, it was decided to replace them.

**Figure 7:**
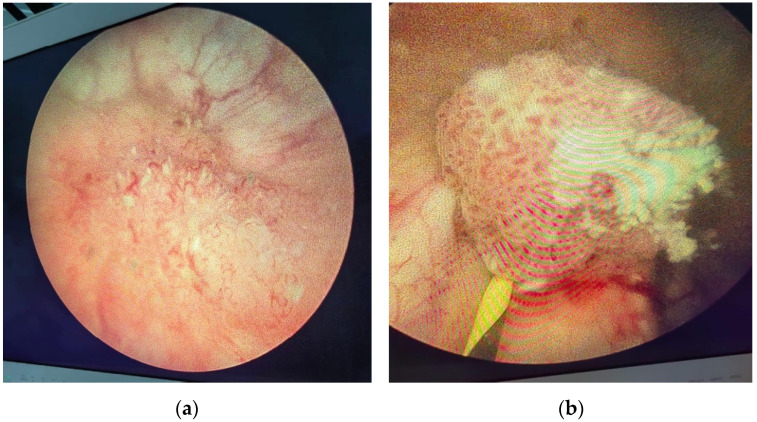
Endoscopic aspect of bladder tumor: (**a**) Bladder tumor located at the level of the right lateral wall. (**b**) Bladder tumor located in the left ureteral orifice.

A cystoscopy under local anesthesia revealed three tumor formations in the bladder: one located at the bladder trigone, measuring approximately 10/5 mm, another on the right lateral wall, and a third at the left ureteral orifice, measuring 10/10 mm (Figure 7).

New bilateral ureteral stents were inserted.

After 3 days in spinal anesthesia, transurethral resection of bladder tumor (TURB) was performed. Intraoperatively, the appearance of the tumor located at the left ureteral orifice was that of a tumor with a starting point from the left ureter (upper urinary tract cell carcinoma).

At 2 days postoperatively (post TURB), contrast CT was performed (creatinine values = 1.39 mg/dL) of the head, thorax, abdomen, and pelvis.

**Figure 8:**
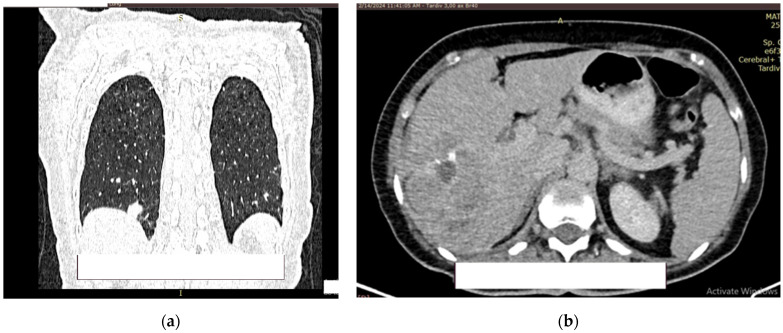
Contrast CT aspect. If there are multiple panels, they should be listed as: (**a**) Description of what is contained in the first panel; (**b**) Description of what is contained in the second panel. Figures should be placed in the main text near to the first time they are cited.

At the level of the pulmonary parenchyma, the CT examination showed multiple pulmonary nodules distributed diffusely and bilaterally with a metastases CT aspect, with the largest located in the posterobasal part of the right lung, with a 14 mm diameter (Figure 8a).

No solid tumor masses were observed in the digestive tract; only intestinal fluid stasis and some hydroaeric levels were present. At the level of the right lobe of the liver, a poly nodular conglomerate with a 98/68 mm maximum diameter in the coronal plane, natively hypodense nodules, and postcontrast with the presence of metal clips from the previous liver surgery were visible (Figure 8b). It was unclear whether this finding represents a secondary liver lesion or is a result of the previous liver metastasectomy.

**Figure 9:**
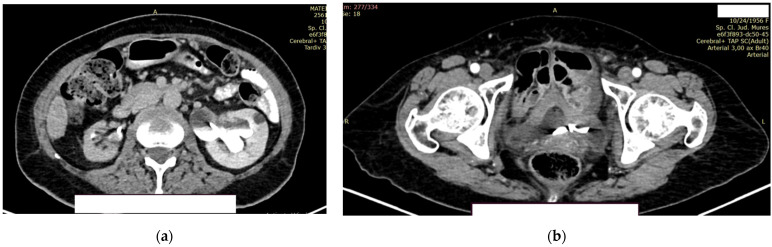
Contrast CT aspect. (**a**) The right kidney is hypotrophic, with a ureteral stent in place, no stasis, and secretion present. The left kidney also has a ureteral stent, with grade II/III hydronephrosis, and both secretion and excretion are present. (**b**) The walls of the urinary bladder are concentrically thickened. It is unclear whether the distal intravesical portion of the ureter has any tumor formation.

The right kidney is hypotrophic, with the presence of ureteral stent, without stones, and without stasis and presence of secretion. The left kidney showed a normal position of the stent, II/III grade hydronephrosis, secretion, and excretion. A retroperitoneal lymph node, measuring up to 10 mm, was observed, with some calcification (Figure 9a).

In the urinary bladder, concentrically thickened walls are described. It cannot be assessed if the distal, intravesical portion of the ureter presents tumors formation (Figure 9b).

**Figure 10:**
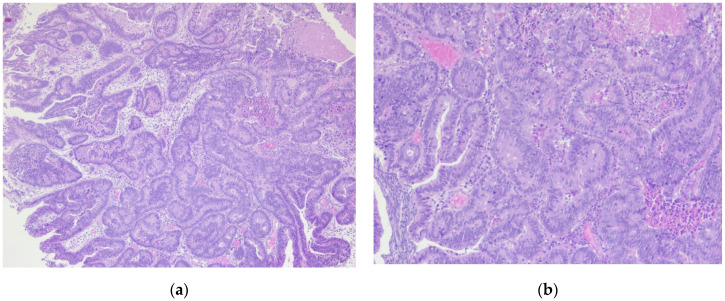
Adenocarcinoma of the urinary bladder: (**a**) The tumor proliferation has a glandular architecture. The appearance is highly suggestive of an infiltrative adenocarcinoma, H&E, 5× magnification. (**b**) The tumor proliferation has a glandular architecture; the glands possess a pseudostratified epithelium with pleomorphic, crowded nuclei, and loss of polarity, H&E, 10× magnification.

The pathological result of the bladder resection showed an infiltrative adenocarcinoma of the bladder without possible specification of whether it is a primary bladder adenocarcinoma or a metastatic colorectal adenocarcinoma (Figure 10). Due to her medical history, this patient chose not to undergo further genetic testing to identify potential genetic alterations.

The future therapeutic strategy of this case is difficult because there are many variables that must be considered. The decision will belong to the oncology committee, a decision that may or may not be accepted by the patient.

## 3. Discussion

Considering the patient’s medical history and current status, some questions arise regarding the future therapeutic attitude:

1. What are the possible risk factors involved in the occurrence of multiple primary tumors? Are there carcinogenic factors that can be managed?
2. Is it a primary bladder adenocarcinoma tumor or metastatic colorectal adenocarcinoma?
3. Can this secondary cancer be treated with curative intent?
4. Is there a possibility for the early diagnosis of these multiple primary tumors?

### 3.1. Risk Factors

The possible factors involved could be viral infection, smoking, genetics, and treatment-related factors.

#### 3.1.1. Viral Factors

Viral infection can be a risk factor for second primary cancer. The main viruses that can be associated with different types of cancer are human papilloma virus (HPV), Epstein–Barr virus (EBV), hepatitis B and C virus, human herpesvirus 8 (HHV-8), and human T-cell lymphotropic virus type 1 (HTLV-1) [5].

Human papilloma virus (HPV)

HPV represents one of the main risk factors for gynecological cancers (cervical, vaginal, and vulvar) [6]. Besides these gynecological cancers, HPV can cause also anal, oropharyngeal, and penile cancer [7].

Prophylactic vaccination against HPV, screening, and treatment of pre-cancer lesions are effective ways to prevent cervical cancer and are cost-effective [8,9]. Besides these gynecological cancers, HPV can cause also anal, oropharyngeal, penile cancer [10,11].

Epstein–Barr virus (EBV)

EBV is considered a risk factor for multiple primary tumors, such as Hodgkin lymphoma, Burkitt lymphoma, nasopharyngeal cancer, gastric cancer, and breast cancer [12,13]. An EBV prophylactic vaccine that induces neutralizing antibodies holds great promise for prevention of EBV-associated diseases [14]. Unfortunately, in 2024, a vaccine against Epstein–Barr virus is not yet available.

Human herpesvirus 8 (HHV-8)

HHV-8 is involved in lymphoma or nasopharyngeal cancer. Despite our increased understanding of HHV-8 pathobiology, the exact mechanisms by which HHV-8 infection causes Kaposi’s sarcoma and lymphoma remain unclear [15,16,17]. There is no systematic progress toward developing an HHV-8 vaccine [18].

Hepatitis B (HBV) and C (HCV)

These viruses are associated in the majority of cases with hepatocellular carcinoma but also other types of tumors (biliary tract cancers, pancreatic cancer, stomach, colorectal, and oral cavity cancer) [19,20]. This association occurs especially with HBV because HBV can be integrated into the host genome, leading to changes in genomic function or chromosomal instability [21,22].

Effective vaccines are available for preventing viral hepatitis B. Effective treatment is also available for people with chronic hepatitis B virus infection. Unlike hepatitis A and B, there is currently no vaccine to prevent hepatitis C infection, but hepatitis C is treated using direct-acting antiviral (DAA) oral medication [23,24].

Human T-cell leukemia virus-1 (HTLV-1)

Even if this oncovirus causes only fatal T-cell leukemia without being a risk factor for other cancers, the discovery of the first pathogenic human retrovirus (HTLV-1) by Gallo in 1979 represented the turning point in demonstrating the oncogenic capacity of other viruses or bacteria [25,26]. There are numerous vaccination research experiments to prevent or control HTLV-1 infection, but no vaccine has been approved by the FDA [27].

#### 3.1.2. Smoking

Smoking increased the overall risk of cancer [28]. Many studies have demonstrated that cigarette smoking is associated with a significantly increased risk of mortality in patients with adenocarcinoma of the colon (CRC) but also in the case of bladder tumors [29,30]. The smoking status can plausibly be considered in the risk stratification of CRC, and smoking cessation can be incorporated into comprehensive treatment planning for patients with CRC or bladder cancer [31].

The effect of smoking on melanoma outcomes remains debatable. Arafa and colleagues [32], in a study on current and heavy smoking, demonstrated that this habit is associated with a higher risk of squamous cell carcinoma (SCC) but a decreased risk of malignant melanoma, while former smoking was not linked to skin cancer risk. In a case-control study published by Sondermeijer et al. [33], a strong inverse association between cigarette smoking and melanoma risk in men was presented.

Smoking is also a risk factor for cervical cancer. Sugawara et al. [34] concluded that there is convincing evidence that cigarette smoking increases the risk of cervical cancer among women. Furthermore, Su et al. [35], in a meta-analysis, provided evidence that passive smoking is associated with an increased risk of cervical cancer

Quitting smoking is very important for cancer survival and to prevent SPM or MPM [36].

#### 3.1.3. Genetics

Lynch syndrome is a mutation of DNA repair genes including the MLH1, MSH2, MSH6, PMS2, and EPCAM genes, which can cause many cancers at a young age. Lynch syndrome carries increased risks of gastrointestinal (especially nonpolyposis colorectal cancer), liver, kidney, brain, and skin cancers [37]. In addition to these tumors, Lynch syndrome is associated with a significant increase in the relative risk of bladder cancer [38].

BRCA gene mutations (especially BRCA1 and BRCA2 genes) [39] are primarily involved in breast and gynecological cancers but are also a risk factor for pancreatic or prostate cancers.

Multiple endocrine neoplasia (MEN1 and MEN2), Li–Fraumeni syndrome, hereditary diffuse gastric cancer syndrome, Peutz–Jeghers syndrome, and PTEN hamartoma tumor syndrome represent other genetic malformations that can cause SPM or MPM [40].

#### 3.1.4. Treatment-Related Factors

Besides their beneficial role for the treatment of multiple types of cancer, chemotherapy and radiotherapy may be associated with an increased risk of secondary primary cancer [41].

##### Chemotherapy

Alkylating agents (mechlorethamine, chlorambucil, cyclophosphamide, melphalan, busulfan, etc.) and platinum-based drugs (cisplatin and carboplatin) are some of the most important drugs used for chemotherapy. However, cyclophosphamide and platinum-based drugs were associated with increased risk of secondary cancer: cyclophosphamide [42] with an increase the risk of leukemia, kidney, and bladder cancers and cisplatin with a risk of leukemia [43].

Vemurafenib and dabrafenib are drugs that target the BRAF (proto-oncogene B-Raf) protein directly. They are especially used to treat melanoma and other cancers. Patients with this type of treatment have a higher risk of squamous cell carcinomas of the skin [44].

Anthracycline topoisomerase II inhibitors include etoposide or VP-16, teniposide, and mitoxantrone.

Topoisomerase inhibitors represent the new potential anti-cancer medications because of their ability to block the normal function of topoisomerases (vital role in DNA replication, transcription, and repair). This will lead to DNA damage and subsequently causes cell death [45]. Unfortunately, one of the long-term effects of this type of treatment is acute leukemia [46].

##### Radiotherapy

Radiotherapy may induce secondary cancers, and this represents a possible side effect of the therapy that must be considered [47]. The incidence of MPMs following radiotherapy has been estimated to be about 5–10% within 10 years [48]. Most studies related to the carcinogenic effect of radiation were conducted on survivors of the atomic bombings in Hiroshima and Nagasaki [49]. The consequence of exposure to ionizing radiation is single-strand and double-strand DNA breaks. Double-strand DNA breaks can lead to gene mutation and malignant transformation of the irradiated cell [50]. Multiple types of secondary cancers have been described as a long-term consequence of radiotherapy. However, the carcinogenic effect of radiotherapy depends on the administered dose, the patient’s age, and the place of radiotherapy [51,52]. In the case of radiotherapy for genitourinary pathologies, the most common secondary tumors are leukemia and malignancies of the colon, rectum, and bladder [53].

### 3.2. Primary Bladder Adenocarcinoma Tumor vs. Metastatic Colorectal Adenocarcinoma

Primary bladder adenocarcinoma (PBA) is a rare tumor accounting for less than 1% of all malignant vesical tumors [54]. PBA is very aggressive, and most of the time, patients present locally advanced stages or distant metastasis. Therefore, overall survival is worse [55]. Differentiation between metastatic colonic adenocarcinoma (MCA) and PBA it is almost impossible based only on pathological features, but almost a quarter of secondary tumors of the urinary bladder are represented by MCA [56]. For the differentiation of PBA from MCA, antibodies are used, especially β-catenin and e-cadherin but also CK7 and CDX-2 [57,58]. However, much larger studies are needed than those that currently exist for their validation for diagnostic purposes. Considering the patient’s medical history, the differentiation between PBA and MCA is impossible in our case.

### 3.3. Can This Secondary Cancer Be Treated with Curative Intent?

The bladder adenocarcinoma has a poorer clinical outcome than urothelial carcinoma [59], firstly due to the superior aggressiveness of bladder adenocarcinoma and secondly due to the presentation of these patients in more advanced stages compared to those with urothelial carcinoma [60].

For a small number of patients in the non-muscle-invasive tumor stage, the endoscopic intervention of transurethral resection of the bladder tumor can represent the therapeutic solution [61]. Most patients with PBA have muscle-invasive disease upon admission, for which the treatment of choice is radical cystectomy with pelvic lymphadenectomy [62]. In case of MCA, the only treatment is a palliative one.

### 3.4. Early Diagnosis Tests for Multiple Primary Tumors

The primary objective of public health programs in oncology is the early detection of cancer. Identifying cancer at its earlier stages has the potential to reduce both cancer-related morbidity and mortality [63]. According to the 2024 WHO report [64], the annual cancer incidence in the USA is around 1.9 million cases, with 600,000 deaths each year. This creates substantial financial strain on healthcare budgets. Hofmarcher et al. [65] estimated the cancer-related costs in Europe for 2018 to be around EUR 199 billion. This amount encompasses expenses for diagnosis and treatment as well as losses in productivity resulting from cancer morbidity and premature mortality. Early cancer detection not only reduces these costs but, most importantly, saves lives [66].

Cancer screening and early detection play a crucial role, frequently leading to better survival rates and enhanced quality of life for asymptomatic cancer patients [63,67]. Currently, several cancers have screening and early detection programs, with the most recognized being for colorectal, breast, cervical, and prostate cancers. Traditional methods for early diagnosis, such as colonoscopy, mammography, low-dose CT scans, PSA testing, and cervical cytology, are endorsed by specialty guidelines [68]. The number of cancers for which screening programs can be applied is limited. The main obstacles include low compliance, reduced sensitivity of screening methods for early-stage disease, high false-positive rates, and high costs [69,70].

The solution to these challenges may lie in multicancer early detection (MCED) tests. These blood-based screenings are designed to detect multiple types of cancer by relying on circulating tumor DNA shed by early-stage tumors [71], shifting the cancer screening paradigm [72].

The primary goal of MCED tests is to detect early-stage cancer cells well before symptoms arise by analyzing various biological samples and combining with artificial intelligence to simultaneously detect a variety of cancers. These tests are referred to as “liquid biopsies”, which involve the use of biological fluids such as saliva or urine, with blood being the most frequently utilized [73].

Recent research has started to assess the effectiveness of these MCED tests. In the PanSeer study, Chen et al. demonstrated through the DNA methylation approach that cancer can be detected non-invasively up to four years earlier than with current standard care methods [74]. The effectiveness of the MCED blood test was further evaluated in the PATHFINDER study, which included patients without any clinical suspicion of cancer. Among those who had a detected tumor signal, 38% had their cancer diagnosis confirmed through specific follow-up investigations [75].

In a case-controlled observational study, Klein et al. [76] demonstrated that the blood-based test achieved an overall sensitivity of 51.5% and a specificity of 99.5% for detecting cancer signals. The primary challenge identified in the study was the limited detection of early-stage cancers, which is likely due to insufficient circulating tumor DNA release into the bloodstream at early stages of the disease.

This lack of detection of early-stage cancers due to the low levels of circulating tumor DNA, along with the high cost [77], limits the widespread use of MCED tests despite their enormous potential to improve patient prognosis and survival. Additional research is required to standardize these diagnostic techniques for cancer, particularly in the case of multiple primary malignant tumors.

## 4. Conclusions

The future therapeutic strategy of this case is difficult because there are many variables that must be considered.

Identification and assessment of risk factors such as viral infection, radiotherapy, chemotherapy, smoking, and genetics are pivotal in understanding and managing multiple primary malignant tumors. Personalized prevention strategies and screening programs may facilitate the early detection of these tumors, whether synchronous or metachronous.

The difference between primary bladder adenocarcinoma tumor and metastatic colorectal adenocarcinoma will make the difference between curative and palliative treatment options.

The use of multicancer early detection (MCED) blood tests for early diagnosis appears promising. However, additional research is needed to standardize these techniques for cancer detection.

## Data Availability

The data presented in this study are available upon request from the corresponding author. The data are not publicly available due to restrictions.
